# Characteristic profiles among students and junior doctors with specific career preferences

**DOI:** 10.1186/1472-6920-13-125

**Published:** 2013-09-12

**Authors:** Yuko Takeda, Kunimasa Morio, Linda Snell, Junji Otaki, Miyako Takahashi, Ichiro Kai

**Affiliations:** 1 King’s College London School of Medicine, London, UK; 2 Mie University School of Medicine, Tsu, Mie, Japan; 3 McGill University Faculty of Medicine, Montreal, Quebec, Canada; 4 Hokkaido University School of Medicine, Sapporo, Hokkaido, Japan; 5 National Cancer Center, Tokyo, Japan; 6 University of Tokyo, Tokyo, Japan

**Keywords:** Career choice, Medical student, Junior doctor

## Abstract

**Background:**

Factors influencing specialty choice have been studied in an attempt to find incentives to enhance the workforce in certain specialties. The notion of “controllable lifestyle (CL) specialties,” defined by work hours and income, is gaining in popularity. As a result, many reports advocate providing a ‘lifestyle-friendly’ work environment to attract medical graduates. However, little has been documented about the priority in choosing specialties across the diverse career opportunities.

This nationwide study was conducted in Japan with the aim of identifying factors that influence specialty choice. It looked for characteristic profiles among senior students and junior doctors who were choosing between different specialties.

**Methods:**

We conducted a survey of 4^th^ and 6^th^ (final)-year medical students and foundation year doctors, using a questionnaire enquiring about their specialty preference and to what extent their decision was influenced by a set of given criteria. The results were subjected to a factor analysis. After identifying factors, we analysed a subset of responses from 6^th^ year students and junior doctors who identified a single specialty as their future career, to calculate a z-score (standard score) of each factor and then we plotted the scores on a cobweb chart to visualise characteristic profiles.

**Results:**

Factor analysis yielded 5 factors that influence career preference. Fifteen specialties were sorted into 4 groups based on the factor with the highest z-score: “fulfilling life with job security” (radiology, ophthalmology, anaesthesiology, dermatology and psychiatry), “bioscientific orientation” (internal medicine subspecialties, surgery, obstetrics and gynaecology, emergency medicine, urology, and neurosurgery), and “personal reasons” (paediatrics and orthopaedics). Two other factors were “advice from others” and “educational experience”. General medicine / family medicine and otolaryngology were categorized as “intermediate” group because of similar degree of influence from 5 factors.

**Conclusion:**

What is valued in deciding a career varies between specialties. Emphasis on lifestyle issues, albeit important, might dissuade students and junior doctors who are more interested in bioscientific aspects of the specialty or have strong personal reasons to pursue the career choice. In order to secure balanced workforce across the specialties, enrolling students with varied background and beliefs should be considered in the student selection process.

## Background

Medical graduates’ career choices are important to understand because they are key determinants of the medical workforce and thus influence how, where, and when medical care will be delivered. In order to discuss the policy implications of managing numbers of specialists, it is critical to elucidate why some specialties are chosen more than others.

There is a substantial literature reporting the factors affecting career choices among medical students. These factors include demographics [1-5], indebtedness [6-12], career-related beliefs, values and attitudes [5,13-18], personality profiles [5,19,20], and academic performance [21,22]. In 1989, Schwartz et al proposed a “controllable lifestyle (CL)” as one of the major factors that influences career decisions [21], and suggested that CL specialties included anaesthesiology, dermatology, emergency medicine, neurology, ophthalmology, otolaryngology, pathology, psychiatry and radiology. An increasing trend toward CL specialties among medical students and graduates has been demonstrated since then [4,5,11,13,23-27]. In the U.S.A. increased competitiveness to enter specialties in the E-ROAD acronym (emergency medicine, radiology, ophthalmology, anaesthesiology, and dermatology) [28] has been noted and much attention has been paid to their presumably desirable lifestyle and financial security [27]. Consequently, many disciplines have attempted to provide a lifestyle-friendly work environment to improve quality of life for trainees and to attract more medical graduates to non CL careers [8,13,23,26,29]. However, the definition of CL specialties remains elusive. Factors might include working hours, sometimes in conjunction with income or investigators’ priori perceptions [11]. A simple dichotomization of specialty between CL and non CL might conceal important complexities or variation [11].

As Cleland et al. have pointed out [30], much of the research in medical students’ career choice have been carried out in the U.S.A. [4,11-13,15,18,21,23-26,31], where most students are graduate entry (enter medical school after a primary university degree) and anticipate an average educational debt of $100,000 to $150,000 [32]. Indebtedness was reported to affect career preference [3,6,8,11,12] and resulted in shortages of the primary care workforce [12,33,34] due to the wide gap of income between primary care and other specialties [22,23,35,36]. In the U.K., on the other hand, the UK Medical Careers Research Group has been conducting extensive cohort studies of medial graduates involving more than 1/3 of practising NHS doctors who qualified since 1974 [37]. It follows the trend of career preference and progression [38-40], and has reported factors associated with specialty choice. For example, enthusiasm for the specialty was the important factor in career choice of ophthalmology and surgery, while the prospect of good working hours and conditions influenced choosing ophthalmology but much less so in surgery [41,42]. Compared to other specialties, a choice of paediatrics was more influenced by experience of the subject as a student [43]. Studies on difference between early career preference and eventual choice of specialties in same individuals were conducted in the cohort [44-46], and revealed that issues of work-life balance were the single most common factor of changing in career choice. However, inadequate salary was chosen by only 1.2% of respondents as a reason of not pursuing preferred specialty [46].

Specialty choice might also be influenced by factors such as the characteristics of a health care delivery system, the practice opportunities available, or the reimbursement policies of government and other payers [47]. In the U.K., about 90% of medical graduates remain in the NHS at four years after qualification [48] and there is similar job satisfaction score between specialties [49]. The managed care system in the U.S.A. was perceived by medical students and residents to limit access to the health system, cause more conflicts and impair the doctor-patient relationship [50]. The demanding role in time-compressed practice makes primary care less attractive as career for future doctors in the U.S.A. [51,52], while good hours and working condition of general practice in U.K. influences the career choice of medical graduates [42]. This implies that studies in a varied healthcare system would add different insight into specialty preference. Although literature from countries other than U.S.A. and U.K. are available, those studies have tended to focus on a few specific specialties [53-55], gender difference [56], graduates who have already chosen the specialty [57], or conducted with limited participants at a single medical school [55]. Therefore, large scale studies on both students and graduates with a focus on diverse specialties are needed to obtain findings more applicable to countries in which the health system is more equitable and most students enter medical school as undergraduates (directly from secondary school).

In Japan, after 6 years of medical school, there is a two year foundation programme consisting of required and elective clinical rotations [57], during which the final decision of career choice is made. Due to the absence of regulatory mechanisms to generate balanced distribution of workforce, it allows virtually any graduate to obtain the type of specialty training desired regardless of their performance during the foundation years. As a result, there are severe shortages in paediatrics and obstetrics, and the primary care workforce has never been filled [58]. Koike S et al. also reported that there have been trends to a further decline in popularity of general medicine, general surgery and obstetrics/gynaecology, while an increasing number of medical graduates are choosing dermatology and anaesthesiology [57].

In 2008, the Japanese government shifted the healthcare policy from limiting to increasing the number of doctors. Since then, the enrollees in medical schools have increased by 10 to 25%. However, it is not clear whether simply increasing the number of medical graduates will result in a sufficient supply of doctors to fulfill the needs of the population, since disparities among specialties already have been recognized [57,58]. Therefore, finding differences in influencing factors for specialty choice would be pertinent, as it might allow consideration of incentives to enhance the workforce in certain specialties, or focused selection of medical school entrants possessing specified characteristics.

In this study, we conducted a cross-sectional survey among medical students and foundation year doctors in Japan to identify factors that influence specialty preference. Based on the subjective importance of the factors in deciding their specialty of choice, we aimed to categorise the 15 specialties to elucidate if there is such a thing as CL specialties from the respondents’ perspectives, and whether providing a lifestyle friendly environment would be an adequate incentive to choose other specialties.

## Methods

### Questionnaire development

A literature search was conducted using MEDLINE from 1988 to 2008 using the search words “career”, “choice” and “medical education.” While very few papers explicitly used a conceptual framework, several authors employed the ‘theory of reasoned action’. The theory of reasoned action (TRA) illustrates that a person’s behaviour is determined by his/her intention to perform the behaviour, and the intention is shaped by two components; the attitude towards the behaviour (defined by beliefs about outcomes of the behaviour and importance of these outcomes), and subjective norms (beliefs about how people he/she feel close will view the behaviour and motivation to meet expectation of these individuals) [59]. All other variables including demographic variables are categorized as external variables that operate through attitudes and norms. This framework was designed to examine the impact of multiple factors influencing specific behaviour [14,60,61].

In order to structure a questionnaire (see Additional file 1: Appendix), we adopted variables used in the study by Gorenflo et al proposing “model of medical student specialty choice based on the theory of reasoned action” [14]. We chose to use this model as it illustrated the attitudinal and normative influence and effects of external variables on the behaviour of choosing a specialty. In Question 16, we selected variables to reflect the two components of TRA [62]: “behavioural beliefs (beliefs that specialty choice leads to certain outcomes)” such as working hours and attainable lifestyle (items 23-30), and the “normative beliefs (beliefs about whether specific individuals or groups approve or disapprove of the specialty choice” such as advice/expectation of parents, advice from teachers/consultants (items 19-22). As external variables, we included demographic variables (age, gender, marital status with or without children, hometown size and proximity to a large city, previous degree, previous employment, doctor in the family, encounter with a doctor as a role model) in the questionnaire. Other external variables derived from the previous findings in literature [1,2,6,7,11,13-15,21,23],[24] were also addressed in Question 16 (Additional file 1: Appendix, items also listed in Table 1).

**Table 1:** Factor analysis of specialty preferences

	**Factors**				
	**I**	**II**	**III**	**IV**	**V**
I. Fulfilling life with job security (α=.86)					
27_Working hours	**.92**	-.10	-.09	.05	-.07
28_Attainable lifestyle	**.83**	-.06	-.14	.10	-.03
30_Risk of my malpractice law suits	**.76**	-.02	-.02	.02	.00
26_Expected income	**.73**	.08	.09	-.10	-.01
29_Influence of future health care reform	**.63**	.07	.04	.03	.07
23_Job availability	**.57**	.09	.16	.00	-.04
24_Ease of opening practice	**.49**	.02	.13	-.16	.08
II. Bioscientific orientation (α=.70)					
6_Mastering the specialty	.00	**.78**	-.04	-.05	-.09
5_Interest in the surgical procedures or technologies	-.06	**.63**	.02	-.04	-.05
2_Interest in the organ specialty	-.04	**.55**	-.04	.02	.04
4_Interest in the research or scientific aspects	.00	**.54**	-.08	.06	.06
9_Prospect for further development of the field	.09	**.53**	-.01	.05	.01
10_Highly respected in society	.04	**.44**	.10	.03	.10
III. Advice from others (α=.82)					
20_Advice from senior students/residents	-.04	-.04	**.90**	.00	-.07
21_Advice from teachers/consultants	-.06	.03	**.75**	.13	-.08
22_Influence of friends	.09	-.04	**.61**	-.01	.06
19_Advice/Expectation of parents	.14	-.04	**.48**	-.08	.16
IV. Educational experience (α=.79)					
15_Received excellent teachings	.00	-.04	.01	**.93**	.00
14_Memorable experience at a class or clinical rotation	-.06	.04	.00	**.80**	.07
16_Comfortable atmosphere at the specialty department	.08	.05	.07	**.64**	-.04
V. Personal reasons (α=.71)					
12_Friend/family suffer(ed) from the illness of the specialty	-.02	.01	-.02	.03	**.77**
11_I suffer(ed) from the illness of the specialty	.02	-.04	-.04	-.01	**.74**
13_Became interested in the specialty before medical school	-.02	.04	.03	.03	**.56**
Inter-factor Correlations	I	II	III	IV	V
I	1.00				
II	.20	1.00			
III	.53	.28	1.00		
IV	.30	.42	.39	1.00	
V	.41	.23	.44	.32	1.00
Excluded items from factor analysis because of;					
Ceiling effect					
1_Interest in the clinical work of the specialty; 8_I feel it rewarding to work in the specialty;					
17_Encounter with role model teachers					
Floor effect					
25_Expectation to inherit practice of my parents/relatives					
Low factor loading (<0.35)					
3_Interest in the targeted populations such as children or the elderly;					
7_I have an aptitude for the specialty					
Correlation with 2 factors to the same extent					
18_Encounter with role model junior doctors					

Factor analysis identified 5 factors that influence career preference on the basis of a set of variables in a questionnaire conducted in 4th and 6th year medical students as well as junior doctors. A principal factor analysis and promax rotation was used since inter-correlations between possible factors were expected from literature and our preliminary analysis. Among 30 items in the questionnaire; 7 items were excluded because of the reasons described at the bottom. Cronbach’s alpha coefficiencies demonstrated internal consistency ranged between 0.70 and 0.86.

We asked participants to respond to items in Question 16 using a four-point scale to rate to what extent the attributes matched their reason for choosing their career specialties (1=not at all; 2=not particularly; 3=fairly well; 4=extremely well).

Participants were asked to specify which of the following 14 medical specialties they intended to pursue; general medicine/family medicine, internal medicine subspecialty, surgery, paediatrics, obstetrics/gynaecology, psychiatry, anaesthesiology, emergency medicine, dermatology, orthopaedics, ophthalmology, otolaryngology, urology and radiology, or “other”. They were instructed to choose one as the most probable specialty and other specialties ‘under consideration’, as many as applied. When “other” was chosen for a non-listed specialty (e.g. neurosurgery), respondents were asked to specify which discipline they were choosing.

We first distributed the survey to content experts to verify comprehensiveness and appropriateness of questionnaire items, then to a small number of students (n=5) and residents (n=5) in two different hospitals as a pilot and to establish face validity. (Additional file 1: Appendix 1)

### Participants and questionnaire administration

A questionnaire survey was conducted anonymously in 4^th^ and 6^th^ year medical students as well as foundation year doctors. Of 80 medical schools in Japan, 49 agreed to participate for 4^th^ year students and 41 for 6^th^ year students. During the three month survey period, 41 and 21 medical schools returned the questionnaire for 4^th^ and 6^th^ year students respectively (4^th^ year students; n=3089, response rate 80.3%, 6^th^ year students; n=1370 and response rate 69.9%). The questionnaire was distributed and collected by the school faculty or an administrator (January to March 2008). In terms of junior doctors, of 849 teaching hospitals in Japan, 342 hospitals participated (n=5320 junior doctors) and 2740 responses were obtained (response rate 51.5%) during the survey period (December 2008 to February 2009). Schools and hospitals which did not return completed questionnaires received up to three reminders by a letter, telephone and facsimile. Individual responses were anonymous, and questionnaire completion was voluntary.

### Data analysis

The factor analysis was conducted in SPSS using a principal factor analysis and promax rotation. In each questionnaire item, we calculated the mean and standard deviation and items showing ceiling effect or floor effect were excluded from the analysis. In order to decide the number of factors, a scree plot was generated. Eigenvalue was set to be greater than 1 and items having a factor loading less than 0.35 or showing a similar factor loading in more than 2 factors were excluded, then the factor analysis was repeated. We calculated the Cronbach’s alpha coefficient for each factor to determine its scale reliability and calculated a mean score and standard deviation.

After the factor analysis, we extracted 6^th^ year students and junior doctors, as they had completed clinical rotations at medical school that might have allowed respondents to obtain deeper insights about specialties. In order to compare and contrast characteristic profiles of respondents considering one particular specialty, we identified respondents who chose a single specialty as the most probable career in this survey (n=2325). We grouped the data under 15 different specialties, and calculated the mean for each factor and the z-score (standard score) from the mean and standard deviation of all valid responses in this survey.

$$z‒\mathrm{score}=50+10x\;\left(x‒\mu \right)/\sigma$$

x: mean score of a factor among senior students and junior doctors who selected a single specialty as the most probable career

μ: mean of the factor obtained from all valid response

σ: standard deviation of the factor obtained from all valid response

This formula implies that if the mean of a factor of the group is equal to the mean of the whole, the z-score is 50. The higher the z-score, the more the influence of the factor in choosing the specialty compared to other factors within the specialty or in other specialties. Our method enables us to visually grasp which factor is more valued by students/junior doctors who chose a certain specialty relative to their peers, across the broad range of specialties. Since we were unable to identify preceding literature using z-score in career preferences, we discuss the face validity of this method by comparing our results with findings from other studies.

We plotted the z-score on a cobweb chart to visualise the characteristic pattern of the respondents choosing a career from broad range of specialties, then grouped the specialties according to the factor with the highest z-score when the difference between the highest and the lowest was significant (>1 SD).

We received ethics approval from the Institutional Review Board of Mie University School of Medicine.

## Results

### Respondents and specialty choice

The respondents of this survey account for 40% of all 4^th^ year and 18% of all 6^th^ year medical students in Japan during that academic year. For the survey of medical graduates in a foundation year programme, we received replies from 18% of residents nationwide. Thirty three to 34% of respondents in each year were female, which reflects the ratio of the target population. Of all respondents, 6626 provided complete responses to the questionnaire items (valid response rate 92.0%); only these were used in factor analysis (4^th^ year students; n=2815, 6^th^ year students; n=1288, residents; n=2523). As well as the 14 given specialties, neurosurgery was listed the most common “other” choice by participants. The number of respondents among 6^th^ year students and junior doctors who identified one of the 15 specialties as “a single most probable specialty of choice” was 2,325. Others chose several specialties with equivalent possibilities.

### Factor analysis

Factor analysis yielded 5 factors that included 23 of the 30 items listed (Table 1); 3 items were excluded due to a ceiling effect; 1 item was excluded due to a floor effect; 2 items were excluded because of low loading factors; one was excluded as it correlated with 2 factors to the same extent.

We defined the following 5 factors based on the types of items that grouped together (Table 1).

Factor 1: Fulfilling life with job security

Factor 2: Bioscientific orientation

Factor 3: Advice from others

Factor 4: Educational experience

Factor 5: Personal reasons

These five factors explained 50.3% of the variance in responses. We calculated Cronbach’s alpha coefficiencies which demonstrated internal consistency that ranged between 0.70 and 0.86.

### Characteristic profile of specialties expressed in cobweb chart

**Figure 1:**
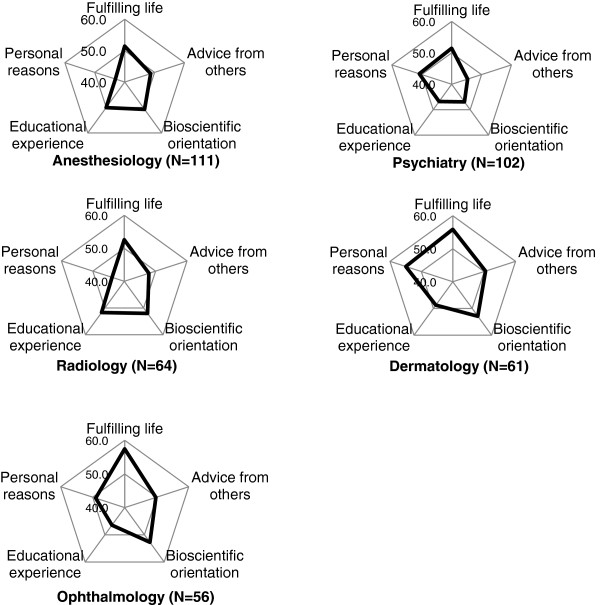
**Fulfilling-life oriented group.** The 6th year medical students and residents who chose one of the specialties in this group as the single most probable specialty or only specialty under consideration showed the highest z-score in the factor of “fulfilling life with job security” compared to other 4 factors, and there was more than 5 (=1 SD) difference between the highest and the lowest scores. The z-score was calculated from the mean of each factor in the specialty, and mean and SD of all valid responses in this survey. The z-score of 50 indicates that the mean of the factor is average of the whole group. The higher the z-score, the more the influence of the factor in choosing the specialty compared to other factors within the specialty or in other specialties.

**Figure 2:**
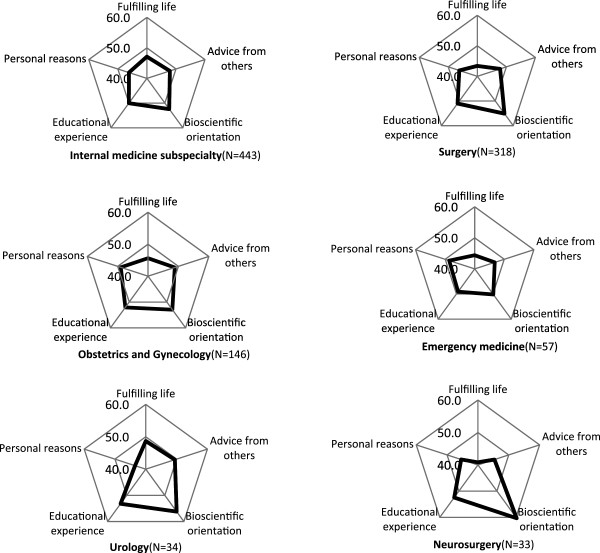
**Bioscientific-orientation group.** Respondents who chose one of these specialties as the most probable career showed higher z-score in factor of bioscientific-orientation compared to other 4 factors. The “fulfilling life” factor ranked second lowest in internal medicine subspecialty and the lowest in other specialties in this group. At the same time, z-score of “educational experience” was ranked second highest among specialties in this group.

**Figure 3:**
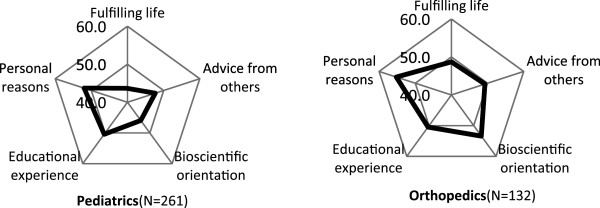
**Personal-reasons group.** Respondents who chose paediatrics or orthopaedics as the most probable career showed the highest z-score in factor of “personal reasons” and the lowest score in “fulfilling life” factor with more than 1SD difference between the two.

**Figure 4:**
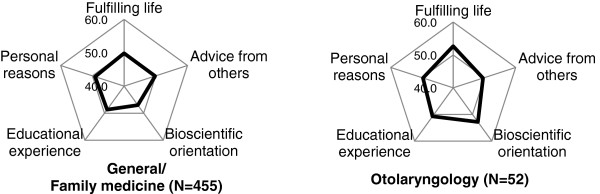
**Intermediate group.** The difference of the highest and lowest z-scores among 5 factors was less than 5 (=1SD) in the respondents choosing general medicine/family medicine or otolaryngology as the most probable career. Because of the similar weight of the 5 factors, General/family medicine and otolaryngology were both categorized as an “intermediate group”. In respondents who considered general medicine/family medicine as their future specialty, the z-scores of all 5 factors were less than 50, which implies external variables relevant to primary care preference were missing in the questionnaire.

The fifteen specialties were classified into 4 groups according to the pattern of z-score. The z-score of “fulfilling life with job security” was the highest compared to other 4 factors in radiology, ophthalmology, anaesthesiology, dermatology and psychiatry (Figure 1). The factor of “bioscientific orientation” was the highest in internal medicine subspecialty, surgery, obstetrics and gynaecology, emergency medicine, urology, and neurosurgery (Figure 2). In paediatrics and orthopaedics, the factor of “personal reasons” showed the highest z-score (Figure 3). In latter 2 groups, z-score of “fulfilling life with job security” was the lowest or the second lowest. The difference between the highest and the lowest z-score in general/family medicine and otolaryngology was less than 1 SD, and we categorised these two as “intermediate group” (Figure 4).

## Discussion

This nation-wide study in Japan identified 5 factors associated with career preferences among medical students and junior doctors, elucidated which factors they valued more in choosing their careers, and compared them to their peers using z-factors. The cobweb chart plotting the z-factors helped us visualise the degree of importance of the factors among trainees with single career interest; choosing one of the 15 different specialties. Although the categorisation of specialties into 4 groups was simply based on the highest and the lowest z-score in each specialty, the patterns of the groups illustrates that there are characteristic profiles among groups.

### “Controllable lifestyle specialties” from future workforce perspectives

Our results confirmed that students/graduates who preferred ROAD specialties (radiology, ophthalmology, anaesthesiology and dermatology) did value the “fulfilling life with job security” factor more than others. While the controllable life style (CL) specialties were originally defined based on the physician’s control of time spent on professional responsibilities [21], the E(emergency medicine)-ROAD specialty was defined by students mainly due to its work hours and income [28]. In our study, the “fulfilling life with job security” factor consists of not only working hours and income, but also job security including risk of malpractice law suits, job availability and influence of future health care reform.

Among the specialties preferred by respondents with the highest z-score in “fulfilling life with job securities” factor, psychiatry was included; however, emergency medicine, often categorized as CL specialty in the U.S., was not in the group. This probably reflects different working conditions in Japan, with a heavy work load in many emergency medicine settings [63].

### Factors that influence preference for “non CL specialties”

Our study further revealed what factors had an influence on choosing a specialty other than CL specialties. The factor of “bioscientific orientation” influenced respondents who preferred one of following 6 specialties; internal medicine subspecialty, surgery, obstetrics/gynaecology, emergency medicine, urology and neurosurgery. This implies that exposing students and residents to the expertise of the discipline, including technologies and research, might enhance the attractiveness of the field. On the other hand students and residents considering paediatrics and orthopaedics were influenced more by the factor “personal reasons”. This factor consists of illness experience and existing interest before entering medical school, which might be identified through interviewing candidates in enrolment process. The “fulfilling life” factor ranked second lowest in internal medicine subspecialty and the lowest in other specialties of “bioscientific orientation” group and “personal reasons” group. At the same time, “educational experience” was ranked second highest in those 2 groups except for orthopaedics ranking it as the 3^rd^. This suggests that when recruiting to a specialty, emphasis on controllable lifestyle rather than learning experience could deter candidates from these fields.

### Expected income as a part of fulfilling life

Newton et al. [11] identified lifestyle and income as separate factors influencing career choice, and reported that students who chose surgery, obstetrics/gynaecology, orthopaedics, and internal medicine subspecialties considered income more important than lifestyle. In our analysis, however, expected income was included in the 7 items forming “fulfilling life with job security” factor (α=0.86). This factor was considered the least important in surgery, obstetrics/gynaecology, orthopaedics and second least important in internal medicine subspecialties. We speculate that this difference reflects the fact that income disparities among specialties are generally not great in Japan due to a wage system based mainly on number of years after graduating from medical school, although wage difference might exist between hospitals or regions. Indebtedness also has not been a major issue in Japan, since most students enter medical school as undergraduates with their parents’ financial support, whereas medical students’ debt has been a policy concern in the U.S. as a cause of shortages in primary care workforce [10].

### Primary care preference as an intermediate group

Since the problem of doctor shortage in primary care in Japan exists even without income disadvantage or financial pressure among medical graduates, simply financing medical education to address students’ debt, or providing financial incentives might not be sufficient to expand the primary care workforce.

General/family medicine and otolaryngology were both categorized as an “intermediate group” because of the similar weight of the 5 factors. However, unlike respondents with an otolaryngology preference, in respondents who considered general/family medicine as their future specialty, the z-scores of all 5 factors were less than 50. This implies we may have missed external variables relevant to a primary care preference. Murdoch et al. reported positive correlation between students’ interest in primary care and the factor of biosocial orientation, including importance of developing long-term patient relationships and enjoyment of tending to patients’ social and psychological needs [15], items which were not included in our questionnaire. On the other hand, in respondents with an interest in general/family medicine, the z-score of “bioscientific orientation” was the lowest among 5 factors. Murdoch et al. showed a negative correlation between bioscientific orientation factor and primary care preference. Generalist career selection was also reported associated with attitudes favouring helping people over opportunities for leadership, intellectual challenge and research [3]. Our results support these findings indirectly affirm the influence of biosocial orientation in potential primary care workforce.

### Study limitations

We developed a questionnaire by implementing items previously utilised in the literature applying a conceptual framework based on the theory of reasoned action (TRA). We did not employ the scale to measure evaluation of beliefs separately from behaviour beliefs according to TRA, however, our results confirmed that subjective norm was one of factors for career choice and there are groups of beliefs that affect the intention. Although the results collectively demonstrate acceptable internal consistency, additional variables focusing on biosocial orientation may have improved its validity especially elucidating preference in primary care.

Regarding the categorization of specialties, we used the z-score and cob-web chart to simply illustrate the impact of influencing factors for career choice and illustrate the differences across the broad range of specialties. Since we were unable to identify literature using z-score for the aforementioned purpose, we had to assume face validity from the similarity in CL and NCL specialties ascertained in this research and existing studies. Diverse profiles according to specialties that were expressed with z-scores suggest that we should consider selecting medical students with varying beliefs and backgrounds that may reflect varied external variables. This may result in a more balanced work force. Further research concurrently analysing demographic variables obtained in this survey would be helpful to identify medical school applicants with interests in a certain specialty. Comparison between students and junior doctors also would be imperative to elucidate changes in perceptions and attitudes with clinical exposures. Those findings could be used to formulate effective interventions at enrolment and during further education to build workforce in need.

Because this is a cross-sectional survey and results were analysed with regard to self-reported specialty preference not based on actual choice, further research is required to demonstrate predictive validity.

## Conclusion

Factors valued by individual junior doctors and senior medical students varied according to their interest in or choice of 15 specialties. Consideration of the characteristic profiles among students and junior doctors with a specific career preference, in order to secure a balanced workforce across the specialties, might include selecting students with various background and beliefs. Emphasis on lifestyle issues, albeit important, might deter some students and junior doctors who are more interested in bioscientific aspects of the specialty or have strong personal reasons toward the career choice. On the other hand, emphasizing only a bioscientific approach might dissuade those interested in primary care. There was significant overlap in factors associated with perceived controllable lifestyle (CL) specialties, a finding similar to studies from the U.S.A. To the best of our knowledge, this is the first nationwide study to survey both medical students and graduates to identify factors influencing their career choice. The results will be useful to those considering interventions to influence career choice and manage the medical workforce.

### Ethical approval

This study was approved by the Institutional Review Board of Mie University School of Medicine.

## Competing interest

The authors declare that they have no competing interests.

## Authors’ contribution

YT served as principal investigator and was responsible for the research design, ethics approval and authorship of the manuscript. KM assisted in all steps of the project and was a major contributor of data collection and analysis. JO and LS are experts in medical education who contributed to research design, interpretation of data and manuscript revision. MT and IK were also intimately involved in research design including questionnaire development, analysis strategy and data interpretation. All authors read and approved the final manuscript.

## Pre-publication history

The pre-publication history for this paper can be accessed here:

http://www.biomedcentral.com/1472-6920/13/125/prepub

## Supplementary Material

Additional file 1: Appendix Questionnaire developed for 4^th^ and 6^th^ year medical students. In questionnaire distributed among junior doctors, 3 items were added: graduated medical school, postgraduate year (PGY 1 or 2), and the size of area where their hospital is located. Click here for file

## References

[B1] Wright B Career choice of new medical students at three Canadian universities: family medicine versus specialty medicine CMAJ 2004 170 13 1920 1924 10.1503/cmaj.1031111 15210640 PMC421719

[B2] Ohtaki J Specialty choice and understanding of primary care among Japanese medical students Med Educ 1996 30 5 378 384 10.1111/j.1365-2923.1996.tb00851.x 8949479

[B3] Kassebaum DG Szenas PL Schuchert MK Determinants of the generalist career intentions of 1995 graduating medical students Acad Med 1996 71 2 198 209 10.1097/00001888-199602000-00030 8615940

[B4] Lambert EM Holmboe ES The relationship between specialty choice and gender of U.S. medical students, 1990-2003 Acad Med 2005 80 9 797 802 10.1097/00001888-200509000-00003 16123456

[B5] Wigney T Parker G Factors encouraging medical students to a career in psychiatry: qualitative analysis Aust N Z J Psychiatry 2008 42 6 520 525 10.1080/00048670802050637 18465379

[B6] Kassebaum DG Szenas PL Relationship between indebtedness and the specialty choices of graduating medical students Acad Med 1992 67 10 700 707 10.1097/00001888-199210000-00018 1388538

[B7] Kibbe MR Effect of educational debt on career and quality of life among academic surgeons Ann Surg 2009 249 2 342 348 10.1097/SLA.0b013e318195e5c8 19212192

[B8] DeZee KJ Effect of financial remuneration on specialty choice of fourth-year U.S. medical students Acad Med 2011 86 2 187 193 10.1097/ACM.0b013e3182045ec9 21169785

[B9] Vanasse A Attractiveness of family medicine for medical students: influence of research and debt Can Fam Physician 2011 57 6 e216 e227 21673198 PMC3114693

[B10] Greysen SR Chen C Mullan F A history of medical student debt: observations and implications for the future of medical education Acad Med 2011 86 7 840 845 10.1097/ACM.0b013e31821daf03 21617506

[B11] Newton DA Grayson MS Thompson LF The variable influence of lifestyle and income on medical students’ career specialty choices: data from two U.S. medical schools, 1998-2004 Acad Med 2005 80 9 809 814 10.1097/00001888-200509000-00005 16123458

[B12] Grayson MS Newton DA Thompson LF Payback time: the associations of debt and income with medical student career choice Med Educ 2012 46 10 983 991 10.1111/j.1365-2923.2012.04340.x 22989132

[B13] Schwartz RW The controllable lifestyle factor and students’ attitudes about specialty selection Acad Med 1990 65 3 207 210 10.1097/00001888-199003000-00016 2306321

[B14] Gorenflo DW Ruffin MT Sheets KJ A multivariate model for specialty preference by medical students J Fam Pract 1994 39 6 570 576 7798861

[B15] Murdoch MM Evaluating the psychometric properties of a scale to measure medical students’ career-related values Acad Med 2001 76 2 157 165 10.1097/00001888-200102000-00015 11158837

[B16] Hauer KE Factors associated with medical students’ career choices regarding internal medicine JAMA 2008 300 10 1154 1164 10.1001/jama.300.10.1154 18780844

[B17] Scott IM Choosing a career in surgery: factors that influence Canadian medical students’ interest in pursuing a surgical career Can J Surg 2008 51 5 371 377 18841235 PMC2556546

[B18] Schwartz MD Medical student interest in internal medicine. Initial report of the society of general internal medicine interest group survey on factors influencing career choice in internal medicine Ann Intern Med 1991 114 1 6 15 1983935 10.7326/0003-4819-114-1-6

[B19] Ciechanowski PS Using relationship styles based on attachment theory to improve understanding of specialty choice in medicine BMC Med Educ 2006 6 3 10.1186/1472-6920-6-3 16405723 PMC1373627

[B20] Petrides KV McManus IC Mapping medical careers: questionnaire assessment of career preferences in medical school applicants and final-year students BMC Med Educ 2004 4 18 10.1186/1472-6920-4-18 15461786 PMC524180

[B21] Schwartz RW Controllable lifestyle: a new factor in career choice by medical students Acad Med 1989 64 10 606 609 10.1097/00001888-198910000-00012 2789604

[B22] Patel MS Katz JT Volpp KG Match rates into higher-income, controllable lifestyle specialties for students from highly ranked, research-based medical schools compared with other applicants J Grad Med Educ 2010 2 3 360 365 10.4300/JGME-D-10-00047.1 21976084 PMC2951775

[B23] Dorsey ER Jarjoura D Rutecki GW Influence of controllable lifestyle on recent trends in specialty choice by US medical students JAMA 2003 290 9 1173 1178 10.1001/jama.290.9.1173 12952999

[B24] Dorsey ER Jarjoura D Rutecki GW The influence of controllable lifestyle and sex on the specialty choices of graduating U.S. medical students, 1996-2003 Acad Med 2005 80 9 791 796 10.1097/00001888-200509000-00002 16123455

[B25] Boyd JS Emergency medicine career choice: a profile of factors and influences from the Association of American Medical Colleges (AAMC) graduation questionnaires Acad Emerg Med 2009 16 6 544 549 10.1111/j.1553-2712.2009.00385.x 19344453

[B26] Schwartz RW Career change: in quest of a controllable lifestyle J Surg Res 1989 47 3 189 192 10.1016/0022-4804(89)90105-4 2770274

[B27] van der Horst K Residents’ reasons for specialty choice: influence of gender, time, patient and career Med Educ 2010 44 6 595 602 10.1111/j.1365-2923.2010.03631.x 20604856

[B28] Barbieri RL EasyROAD-high road or path of least resistance? OBG Management 2004 16 December 8 9

[B29] Hauer KE Internal medicine clerkship directors’ perceptions about student interest in internal medicine careers J Gen Intern Med 2008 23 7 1101 1104 10.1007/s11606-008-0640-y 18612752 PMC2517945

[B30] Cleland J Associations between medical school and career preferences in Year 1 medical students in Scotland Med Educ 2012 46 5 473 484 10.1111/j.1365-2923.2012.04218.x 22515755

[B31] Bland CJ Meurer LN Maldonado G Determinants of primary care specialty choice: a non-statistical meta-analysis of the literature Acad Med 1995 70 7 620 641 10.1097/00001888-199507000-00013 7612128

[B32] Chen DC Characterizing changes in student empathy throughout medical school Med Teach 2012 34 4 305 311 10.3109/0142159X.2012.644600 22455699

[B33] Schwartz MD Changes in medical students’ views of internal medicine careers from 1990 to 2007 Arch Intern Med 2011 171 8 744 749 10.1001/archinternmed.2011.139 21518941

[B34] Palmeri M Economic impact of a primary care career: a harsh reality for medical students and the nation Acad Med 2010 85 11 1692 1697 10.1097/ACM.0b013e3181f5b754 20881829

[B35] Sommers BD Bindman AB New physicians, the affordable care act, and the changing practice of medicine JAMA 2012 307 16 1697 1698 10.1001/jama.2012.523 22535852

[B36] Tanne JH Income and job satisfaction fall among US doctors BMJ 2012 344 e3109 10.1136/bmj.e3109 22549071

[B37] UK Medical Careers Research Group Cohort studies of doctors’ careers[cited 2012 8 August]; Available from: http://www.uhce.ox.ac.uk/ukmcrg/24021778

[B38] Lambert TW Goldacre MJ Turner G Career choices of United Kingdom medical graduates of 2002: questionnaire survey Med Educ 2006 40 6 514 521 10.1111/j.1365-2929.2006.02480.x 16700766

[B39] Svirko E Goldacre MJ Lambert T Career choices of the United Kingdom medical graduates of 2005, 2008 and 2009: questionnaire surveys Med Teach 2013 35 5 365 375 10.3109/0142159X.2012.746450 23360485

[B40] Goldacre MJ Lambert TW Laxton L Career choices made for the hospital medical specialties by graduates from UK medical schools, 1974-2005 Clin Med 2009 9 1 42 48 10.7861/clinmedicine.9-1-42 19271600 PMC5922632

[B41] Lambert TW Goldacre MJ Bron AJ Career choices for ophthalmology made by newly qualified doctors in the United Kingdom, 1974-2005 BMC Ophthalmol 2008 8 3 10.1186/1471-2415-8-3 18318905 PMC2311274

[B42] Goldacre MJ Early career choices and successful career progression in surgery in the UK: prospective cohort studies BMC Surg 2010 10 32 10.1186/1471-2482-10-32 21044317 PMC2987756

[B43] Turner G Career choices for paediatrics: national surveys of graduates of 1974-2002 from UK medical schools Child Care Health Dev 2007 33 3 340 346 10.1111/j.1365-2214.2006.00664.x 17439449

[B44] Goldacre MJ Lambert TW Stability and change in career choices of junior doctors: postal questionnaire surveys of the United Kingdom qualifiers of 1993 Med Educ 2000 34 9 700 707 10.1046/j.1365-2923.2000.00667.x 10972747

[B45] Goldacre MJ Laxton L Lambert TW Medical graduates’ early career choices of specialty and their eventual specialty destinations: UK prospective cohort studies BMJ 2010 341 c3199 10.1136/bmj.c3199 20605892 PMC2897977

[B46] Goldacre MJ Goldacre R Lambert TW Doctors who considered but did not pursue specific clinical specialties as careers: questionnaire surveys J R Soc Med 2012 105 4 166 176 10.1258/jrsm.2012.110173 22532656 PMC3343714

[B47] Barzansky B Commentary: Research on specialty choice: the challenge is in the details Educ Health (Abingdon) 2000 13 2 197 200 10.1080/13576280050074453 14742079

[B48] Goldacre M Davidson J Lambert T The junior doctor exodus BMJ Careers 20 October 2010. http://careers.bmj.com/careers/advice/view-article.html?id=20001543

[B49] Davidson JM UK senior doctors’ career destinations, job satisfaction, and future intentions: questionnaire survey BMJ 2002 325 7366 685 6 10.1136/bmj.325.7366.685 12351361 PMC126656

[B50] Simon SR Views of managed care–a survey of students, residents, faculty, and deans at medical schools in the United States N Engl J Med 1999 340 12 928 36 10.1056/NEJM199903253401206 10089187

[B51] Whitcomb ME Cohen JJ The future of primary care medicine N Engl J Med 2004 351 7 710 2 10.1056/NEJMsb045003 15306674

[B52] Lee TH Perspective roundtable: redesigning primary care N Engl J Med 2008 359 20 e24 10.1056/NEJMp0809050 19005187

[B53] Henderson E Berlin A Fuller J Attitude of medical students towards general practice and general practitioners Br J Gen Pract 2002 52 478 359 63 12014532 PMC1314290

[B54] Sobral DT Selective training and cross-year clinical tutoring as educational influences on generalist career choice Educ Health (Abingdon) 2001 14 2 295 303 10.1080/13576280110051028 14742028

[B55] Avan BI Factors influencing the selection of surgical specialty among Pakistani medical graduates J Postgrad Med 2003 49 3 197 200 discussion 201 14597779

[B56] Chang PY Factors influencing medical students’ choice of specialty J Formos Med Assoc 2006 105 6 489 96 10.1016/S0929-6646(09)60189-3 16801037

[B57] Koike S Postgraduate training and career choices: an analysis of the National Physicians Survey in Japan Med Educ 2010 44 3 289 97 10.1111/j.1365-2923.2009.03582.x 20444060

[B58] Yasunaga H The catastrophic collapse of morale among hospital physicians in Japan Risk Manag Healthc Policy 2008 1 1 6 22312197 10.2147/RMHP.S4379 PMC3270896

[B59] Fishbein M Fishbein M A behavior theory approach to the relations between belief about an object and the attitude toward the object Readings in attitude theory and measurement 1967 New York: John Wiley and Sons, Inc 389 400

[B60] Montano DE A survey of fourth-year medical students’ decisions regarding family practice as a career J Med Educ 1988 63 11 830 8 3184148 10.1097/00001888-198811000-00002

[B61] Chandarana PC Loncke M Conlon P Factors influencing medical students’ intentions to choose psychiatry as a career Can J Psychiatry 1989 34 5 425 9 2766194 10.1177/070674378903400511

[B62] Montano DE Kasprzyk D Glanz K, Rimer BK, Viswanath K Theory of reasoned action, theory of planned behavior, and the integrated behavioral model Health behavior and health education 2008 Wiley: Sab Francisco 67 96

[B63] Okamoto H An occupational health study of emergency physicians in Japan: health assessment by immune variables (CD4, CD8, CD56, and NK cell activity) at the beginning of work J Occup Health 2008 50 2 136 46 10.1539/joh.L6084 18403864

