# Supplementary material for: Characteristic profiles among students and junior doctors with specific career preferences
Source: BMC Med Educ. 2013 Sep 12;13:125. doi: 10.1186/1472-6920-13-125 (PMC3847686; doi:10.1186/1472-6920-13-125)
Supplement: Additional file 1: Appendix — Questionnaire developed for 4th and 6th year medical students. In questionnaire distributed among junior doctors, 3 items were added: graduated medical school, postgraduate year (PGY 1 or 2), and the size of area where their hospital is located. [file 1472-6920-13-125-S1.pdf]

Medical Students

Questionnaire Survey on Career Choice

We are interested in your future career plans. This questionnaire asks what factors are important to you when you decide your specialty as well as the location of your future practice site. We are conducting this survey among medical students nationwide. Your response is used only for the purpose of this study and respondents will never be identified. The results of this survey will be revealed on our home page without any information implying the identity of the respondents nor his/her university.

Please choose the appropriate number and fill out the text boxes provided on the right side of this sheet or circle the relevant choice.

This questionnaire should take 8 minutes to complete. We would be extremely grateful if you would take the time to complete it.

Inquires for details:
Research Group on Disparities of Health Care resources
（Grant: Ministry of Education）
Principle Investigator: Yuko Takeda, MD
Mie University Graduate School of Medicine
Phone 059-231-5620, Facsimile 059-231-5580

I. Please give the following information about yourself.

Text boxes

Q1. Gender 1. Male 2. Female

[Q1:]

Q2. Age

[Q2: y.o.]

Q3. Have you entered any other college or university before medical school?
1. Yes（science course） 2. Yes（liberal arts course） 3. No

[Q3:]

Q4. Do you have any other undergraduate degrees apart from your primary medical degree?
1. Yes（science course） 2. Yes（liberal arts course） 3. No

[Q4:]

Q5. Do you have work experience before medical school)?
1. Yes 2. No

[Q5:]

Q6. Marital status 1. Married 2. Single

[Q6:]

Q7. Do you have children? 1. Yes 2．No

[Q7:]

Q8. Which of the following categories best describes the area you grew up in until 18 years of age? Please pick one on the basis of your overall impression. The number in ( ) gives a rough idea of the size of the area.

1. Large city (with a population of more than 500,000)
2. Midsize city (with a population in the 100,000 to 300,000 range)
3. Small city (with a population of 50,000))
4. Town/Village (smaller municipalities)
5. Remote area/islands (Rural area)

[Q8:]

Q9. Pick one of the following ordinance-designated cities that you felt closest to when you lived in the area that you chose in Q8, and write down how many hours it took to get there by using a popular transportation system at the time.
＜Sapporo、Tokyo、Kawasaki、Yokohama、Nagoya、Kyoto、Osaka、Kobe、Hiroshima、Kita-Kyushu、Fukuoka＞

e. g. Tokyo was felt closest to Naha where I grew up, and it took 3.5 hours by air plane from Naha to Tokyo.

e. g. Grew up in Nagoya ･･･････ Zero hours

[Q9: approx hours]

Q10. Is your mother and/or father a physician or a dentist?

1. Yes 2. No

Q10	

Q11. Before entering a medical school, did you personally know a physician or a dentist with whom you felt very close? (excluding family members who may be physicians or dentists.)

1. Yes 2. No

Q11	

Q12. Before entering a medical school, did you encounter a physician whom you perceived as a role model? (The physician could also be a character in a book or movie.)

1. Yes 2. No

Q12	

II. Future Career Plans in Specialty Choice

Q13. What do you hope to be in future. (circle all that apply.)

1. Clinician -------------------------- **(proceed to Q14)**
2. Basic medicine scientist
3. Scholar in public health
4. Officer at public health institute
5. Governmental officer at the Ministry of Health, Labour and Welfare
6. other
7. Undecided

Q1 (please circle all that apply.)
1. Clinician
2. Basic medicine scientist
3. Scholar in public health
4. Officer at public health institute
5. Governmental officer
6. others
7. Undecided

Q14. **If you chose Clinician in Q13**, please choose one of the following types of physicians to which you feel most accurately describes your goals.

1. Highly specialized physician with strong interest in cutting edge technologies
2. Work with patients to find the best approach to their concerns or healthproblems
3. Either of above is equally applicable or unable to pick one

Q14	

Q15. **If you chose Clinician in Q13**, which specialty are you interested in? (Please circle all that apply., Also, please choose just one that you are currently most interested in and doubled circle this choice ◎)

1. General medicine/family medicine
2. Internal medicine subspecialty
3. Surgery
4. Paediatrics
5. Obstetrics and gynecology
6. Psychiatry
7. Anaesthesia
8. Emergency medicine
9. Dermatology
10. Orthopaedic surgery
11. Ophthalmology
12. Otorhinolaryngology ENT
13. Urology
14. Radiology
15. Other (Please specify)

Q15 (◎: the most interestedspecialty) (○: specialty of interest, circle all that apply)
1.General medicine/family medicine
2.Internal medicine subspecialty
3.Surgery
4.Paediatrics
5.Obstetrics and Gynecology
6.Psychiatry
7.Anaesthesia
8.Ermergency medicine
9.Dermatology
10.Orthopaedic surgery
11.Ophthalmology
12.Otorhinolaryngology
13.Urology
14.Radiology
15.Others ( )

Q16. To what extent do the following reasons for choosing a specialty match your own? Please circle one response for each factor. 〔4. Extremely, 3. fairly well, 2. not particulary 1. Not at all〕	Extremely 4	3	2	Not at all 1
**Characteristics of the specialty**				
1. Interest in the clinical work of the specialty	4	3	2	1
2. Interest in the organ of the specialty	4	3	2	1
3. Interest in the targeted population (e.g. children, the elderly)	4	3	2	1
4. Interest in the research or scientific aspects	4	3	2	1
5. Interested in the surgical procedures or technologies	4	3	2	1
6. Mastering the specialty	4	3	2	1
7. I have an aptitude for the specialty	4	3	2	1
8. I feel it rewarding to work in the specialty	4	3	2	1
9. Prospect for further development of the field	4	3	2	1
10. Highly respected in society	4	3	2	1
**Personal experience**				
11. I suffer(ed) from the illness of the specialty	4	3	2	1
12. Friend/family suffer(ed) from the illness of the specialty	4	3	2	1
13. Became interested in the specialty before medical school	4	3	2	1
**Experience at a medical school or during postgraduate training**				
14. Memorable experience at a class or clinical rotation	4	3	2	1
15. Received excellent teachings	4	3	2	1
16. Comfortable atmosphere at the specialty department	4	3	2	1
17. Encounter with role model teachers	4	3	2	1
18. Encounter with role model junior doctors	4	3	2	1
**Advice from others**				
19. Advice/Expectation of parents	4	3	2	1
20. Advice from senior students/residents	4	3	2	1
21. Advice from teachers/consultants	4	3	2	1
22. Influence of friends	4	3	2	1
**Considering future work condition**				
23. Job availability	4	3	2	1
24. Ease of opening practice	4	3	2	1
25. Expectation to inherit practice of my parents/relatives	4	3	2	1
26. Expected income	4	3	2	1
27. Working hours	4	3	2	1
28. Attainable lifestyle	4	3	2	1
29. Influence of future health care reform	4	3	2	1
30. Risk of my malpractice law suits	4	3	2	1
**Others**				
31. Other（Please describe ）	4	3	2	1

III. Future Career Plans in Practice Location

Q17. When you decide where to practice in the future, to what extent are the following important to you? Please circle one response for each factor. 〔4. very important 3. fairly important 2. not particularly important 1. not at all〕	Very important 4	3	2	Not at all 1
1.Your hometown	4	3	2	1
2.Hometown of partner	4	3	2	1
3.Parents' residence	4	3	2	1
4.Partner's career	4	3	2	1
5.Partner's preference	4	3	2	1
6.Educational environment for children	4	3	2	1
7.Location of your medical school	4	3	2	1
8.Location of teaching hospital where you completed residency	4	3	2	1
9.Career development	4	3	2	1
10. Research environment	4	3	2	1
11. Teaching opportunities	4	3	2	1
12. Availability of nearby specialized hospitals for referrals	4	3	2	1
13. Availability of support from other doctors	4	3	2	1
14. Community atmosphere (temperament and characteristics of people)	4	3	2	1
15. Climate and/or natural environment	4	3	2	1
16. Lifestyle	4	3	2	1
17. Income	4	3	2	1
18. Possibility of inheriting practice of my parents/relatives	4	3	2	1
19. Assignment from your department head	4	3	2	1
20.Others （Please describe ）	4	3	2	1

Q18. To what extent are you willing to work in a rural area (under the condition that your specialty is needed and helpful to the area)?

1. Keenly-motivated
2. Willing to work for certain period of time
3. Would rather avoid
4. Never
5. Others (Please describe)

Q18	

IV. Special Request to all respondents

We would like to send you further questionnaires to follow your career progress. If you are willing to cooperate, please fill the following box with an 8 digit number representing your date of birth. You are not identified by providing this information and this is completely optional.

e.g.: 1985, September 17the→ 1 9 8 5 0 9 1 7

＊＊＊ Thank you very much for your cooperation. ＊＊＊
